# The Standardized Letter of Evaluation: How We Perceive the Quiet Student

**DOI:** 10.5811/westjem.2022.12.56137

**Published:** 2023-02-27

**Authors:** John K. Quinn, Jillian Mongelluzzo, Newton Addo, Alyssa Nip, Joseph Graterol, Esther H. Chen

**Affiliations:** University of California, San Francisco, Department of Emergency Medicine, San Francisco, California

## Abstract

**Introduction:**

The Standardized Letter of Evaluation (SLOE) is an emergency medicine (EM)-specific assessment designed to help EM residency programs differentiate applicants. We became interested in SLOE-narrative language referencing personality when we observed less enthusiasm for applicants described as “quiet” in their SLOEs. In this study our objective was to compare how quiet-labeled, EM-bound applicants were ranked compared to their non-quiet peers in the global assessment (GA) and anticipated rank list (ARL) categories in the SLOE.

**Methods:**

We conducted a planned subgroup analysis of a retrospective cohort study of all core EM clerkship SLOEs submitted to one, four-year academic EM residency program in the 2016–2017 recruitment cycle. We compared SLOEs of applicants who were described as “quiet,” “shy,” and/or “reserved” — collectively referred to as “quiet” — to SLOEs from all other applicants, referred to as “non-quiet.” We compared frequencies of quiet to non-quiet students in GA and ARL categories using chi-square goodness-of-fit tests with a rejection criteria (alpha) of 0.05.

**Results:**

We reviewed 1,582 SLOEs from 696 applicants. Of these, 120 SLOEs described quiet applicants. The distributions of quiet and non-quiet applicants across GA and ARL categories were significantly different (P < 0.001). Quiet applicants were less likely than non-quiet applicants to be ranked in the top 10% and top one-third GA categories combined (31% vs 60%) and more likely to be in the middle one-third category (58% vs 32%). For ARL, quiet applicants were also less likely to be ranked in the top 10% and top one-third categories combined (33% vs 58%) and more likely to be in the middle one-third category (50% vs 31%).

**Conclusion:**

Emergency medicine-bound students described as quiet in their SLOEs were less likely to be ranked in the top GA and ARL categories compared to non-quiet students. More research is needed to determine the cause of these ranking disparities and address potential biases in teaching and assessment practices.

## INTRODUCTION

The Standardized Letter of Evaluation (SLOE) is designed to assist emergency medicine (EM) residency programs to differentiate applicants and is considered very important in the decision to interview a candidate.^1^ The SLOE includes the applicant’s qualifications for EM, a narrative assessment of cognitive and non-cognitive attributes, and the applicant’s rank in GA and ARL categories, as compared to other applicants.

We became interested in personality factors that may put an applicant at a disadvantage when we observed less enthusiasm for applicants described as “quiet” in their SLOE narratives. This reaction was consistent with studies showing that being described as quiet on internal medicine clerkship evaluations was interpreted as a negative attribute or “red flag,” even when the comment was not linked to performance.^2^,^3^ Further, introverted medical students and residents scored lower than extraverts on subjective clinical evaluations but not on objective assessments.^4^,^5^ Although some studies found extraversion to be related to aspects of success in and outside of medical careers,^6^,^7^ others identified more nuanced measures of personality and non-cognitive attributes to be related to success (eg, conscientiousness,^6^,^8^ emotional stability,^8^ and proactivity^9^), qualities possessed by both introverts and extraverts. We found no studies suggesting that quiet individuals were unsuccessful in, or unsuited for, EM careers.

While residency programs strive to reduce bias in assessment and recruitment, there has been little research on how quiet students are perceived or whether a “quiet bias” exists in EM training. We compared the GA and ARL categories in the SLOEs of quiet EM applicants to non-quiet applicants.

## METHODS

### Study Design

We conducted a planned subgroup analysis of a retrospective cohort study of all core EM clerkship SLOEs submitted to one, four-year academic EM residency program in the 2016–2017 recruitment cycle. We excluded SLOEs from a non-Liaison Committee on Medical Education accredited school, and from students who had graduated from medical school during the application cycle. The study was approved by the institutional review board and the Association of American Medical Colleges.

### Study Setting and Population

We compared SLOEs from applicants who were described as “quiet,” “shy,” and/or “reserved” —collectively referred to as “quiet” — to SLOEs from all other applicants, collectively referred to as “non-quiet.” We chose the descriptors “quiet, shy and reserved” because they are typically used to describe introverts.

### Study Protocol

The SLOEs were downloaded from the Electronic Residency Application Service into REDCap electronic data capture tools hosted at University of California, San Francisco by JM and de-identified. Demographic information was self-identified by applicants. Gender identification was mandatory while race and ethnicity were optional. Data from SLOEs was extracted by AN and JG and included geographic region of medical school attended, GA (top 10%, top one-third, middle one-third, lower one-third), ARL (top 10%, top one-third, middle one-third, lower one-third, unlikely to be ranked), and narrative comments.

### Data Analysis

We used descriptive statistics to describe demographic makeup of the study population with percentages where appropriate. We applied Pearson’s chi-square test to compare categorical data using R version 3.6 (The R Foundation for Statistical Computing, Indianapolis, IN) and presented this analysis with *P*-values.

## RESULTS

We reviewed 1,582 SLOES from 696 applicants; 120 SLOEs from 107 applicants included the words “quiet,” “shy”, and/or “reserved” to describe the applicant’s personality. The distribution of quiet and non-quiet applicants was not significantly different across race, gender, and geographic region of medical school attended (Table 1). Neither was there a significant difference between quiet and non-quiet students by the gender of the SLOE writer (Table 2).

The distributions of quiet and non-quiet applicants on GA (*P* <0.001) and ARL (*P* < 0.001) were significantly different (Table 2). For GA, quiet applicants were significantly less likely to be ranked in the top 10% and top one-third categories combined (31% vs 60%) and more likely to be in the middle one-third category (58% vs 32%), compared to non-quiet applicants. Similarly, for ARL, quiet applicants were significantly less likely to be ranked in the top 10% and top one-third categories combined (33% vs 58%) and more likely to be in the middle one-third category (50% vs 31%) compared to non-quiet applicants (Table 2). Finally, we found no difference (P = 0.66) in the discrepancy between GA and ARL categories (Table 2).

## DISCUSSION

Emergency medicine-bound students described in their SLOEs as quiet, shy, and/or reserved were less likely to be ranked in the top GA and ARL categories compared to non-quiet applicants. We found no differences among relationships between quiet applicants and geographic region of medical school, race or ethnicity, gender, or SLOE-writer gender. At face value, this suggests that quiet students may be perceived as less suited for EM clinical settings than non-quiet students. However, other studies have shown that emergency physicians are a heterogenous group with wide-ranging personality attributes and that this diversity may play an important role in team dynamics.^9^,^10^

While we did not assess causality, our findings suggest the need to investigate the possibility that teaching and assessment practices in EM training favor the personality and learning style of extraverts, as shown in other clinical settings.^11^,^12^ For example, teaching methods that include interactive-learning, peer-led discussion, and rapid-response questioning reward extraverts for assertiveness and allow them to overshadow their introverted peers.^11^,^12^ Consequently, evaluators may unfairly perceive introverts as less motivated, knowledgeable, or prepared, which is reflected in poor performance evaluations.^11^ Similarly, assessment criteria that value characteristics of extraverts (eg, initiates and leads discussions) may undervalue the strengths of introverts (eg, synthesizes information, listens before engaging, reflective).^11^,^12^ Medical students who self-identify as introverts report they are aware of the “quiet” bias in medical training and often feel misunderstood and unfairly judged.^12^

Changes to instructional and assessment practices may create a more supportive environment for introverted learners. Instructional changes could include alternating leadership roles, providing reflection time for responses, and offering student-mentorship to help introverts navigate the learning environment.^6^,^11^,^12^ Assessment changes such as increasing evaluator-student observations, using assessment tools that focus on skill acquisition, and referencing personality only as it relates to performance, may result in more equitable assessment.^5^,^12^

The ranking disparity identified in this study has high-stakes implications for quiet, EM-bound students who may be at a disadvantage when competing for residency, and warrants further investigation to determine its cause. Examining teaching and assessment practices in the clinical environment may help identify ways to support quiet students in their medical training.

## LIMITATIONS

This study has several limitations. We reviewed applications submitted to only one EM residency program, from a single recruitment cycle in 2016, which may not reflect current best practices for writing SLOEs. We did not determine the causality of ranking disparities observed in this study, nor did we assess the contribution of other performance measures such as clerkship grades or board scores. Describing students as quiet, shy, or reserved may not reflect their personality, but rather how they were perceived by their evaluator in the clinical setting in which they were observed. Applicants did not receive a personality inventory nor did they self-report their personality type.

## CONCLUSION

Emergency medicine-bound students described as “quiet” in their Standardized Letters of Evaluation were less likely to be ranked in the top global assessment and anticipated rank list compared to non-quiet students. More research is needed to determine its cause and address potential biases in teaching and assessment practices.

## Figures and Tables

**Table 1:** Applicant demographic information.

Self-reported demographics	All applicants [n (%)]	Quiet applicants [n (%)]	Non-quiet applicants [n (%)]	Chi-square (P-value)
Total	696	107	589	
Race				
White	354 (51)	50 (47)	304 (52)	0.56
Asian	157 (23)	24 (22)	133 (23)	
Latinx	56 (8)	10 (9)	46 (8)	
Black	48 (7)	6 (6)	42 (7)	
Other	81 (12)	17 (16)	64 (11)	
Gender				
Male	446 (64)	69 (64)	377 (64)	0.92
Female	250 (36)	38 (36)	212 (36)	
Geographic region of medical school*				
Northeast	164 (24)	24 (22)	140 (24)	0.83
Midwest	158 (23)	28 (26)	130 (22)	
South	191 (27)	28 (26)	163 (28)	
West	183 (26)	27 (25)	156 (26)	

* Categorized according to National Inpatient Sample, (https://www.hcup-us.ahrq.gov/db/nation/nis/NIS_Introduction_2010.jsp#figure2)

**Table 2:** Global assessment and rank list categories for quiet vs non-quiet applicants.

SLOE attributes	All SLOEs [n (%)]	Quiet applicant SLOEs [n (%)]	Non-quiet applicant SLOEs [n (%)]	Chi-square (P-value)
Writer gender				
Male	837 (53)	69 (58)	768 (53)	0.57
Female	550 (35)	38 (32)	512 (35)	
Group	195 (12)	13 (11)	182 (12)	
Global assessment				
Top 10%	325 (21)	11 (9)	314 (21)	<0.001
Top 1/3	599 (38)	26 (22)	573 (39)	
Middle 1/3	531 (34)	69 (58)	462 (32)	
Lower 1/3	127 (8)	14 (12)	113 (8)	
Rank list				
Top 10%	321 (20)	11 (9)	310 (21)	<0.001
Top 1/3	575 (36)	29 (24)	546 (37)	
Middle 1/3	517 (33)	60 (50)	457 (31)	
Lower 1/3	152 (10)	18 (15)	134 (9)	
UTBR	8 (1)	2 (2)	6 (<1)	
No data	9 (1)	0 (0)	9 (1)	N/A
Discrepancy*				
No change	1317 (83)	104 (87)	1213 (83)	0.66
Up	97 (6)	6 (5)	9 (6)	
Down	159 (10)	10 (8)	149 (10)	

* Rank list category changes relative to global assessment (9 SLOEs were missing rank list data).

*SLOE*, Standardized Letter of Evaluation; *N/A*, not applicable; *UTBR*, unlikely to be ranked.
